# Ultrasound can deliver chemical stimulants to the skin and modulate their perception

**DOI:** 10.1038/s41598-025-94463-7

**Published:** 2025-03-25

**Authors:** Tor-Salve Dalsgaard, Arpit Bhatia, Lei Gao, Ryuji Hirayama, Sriram Subramanian, Joanna Bergström, Kasper Hornbæk

**Affiliations:** 1 Department of Computer Science, University of Copenhagen, Universitetsparken 1, 2100 Copenhagen, Denmark; 2 Department of Computer Science, University College London, Euston Road 169, London, United Kingdom

**Keywords:** Acoustophoresis, Topical Stimulants, Haptic Sensations, Computer science, Somatosensory system

## Abstract

When applied to the skin, chemical stimulants can evoke haptic sensations. However, they need to be applied continuously using paper or pads in fixed locations, limiting their usefulness as a general haptic technology. To overcome these limitations, we introduce an ultrasound-based system for the precise acoustophoresis of droplets of chemical stimulants to the skin. We show that such droplets can indeed produce distinct haptic sensations. In addition, the system can use ultrasound to stimulate the area of the skin where the stimulants have been applied. We show that this increases the perceived intensity. Taken together, these results demonstrate the promise of non-contact delivery and modulation of chemical stimulants, not only as a haptic technology but also to provide deeper insights into the interaction of the chemical and mechanical senses.

## Introduction

Chemical stimulants, such as menthol, capsaicin, and cinnamal, can induce haptic sensations when applied to the skin^1–7^. Chemicals have been used to activate the somatosensory system in the mucosal skin regions (like the mouth, eyelids, and nostrils)^8^, inducing sensations of itching, warmth, and coolness^7,9,10^, as well as in non-mucosal areas of the body with thin epidermis layers, such as the volar forearm^3,6,7^ and the lips^11^.

The most common way to deliver the chemicals to soak a sheet of filter paper or a cotton pad with the chemical and place it on the skin^1–7^. However, these delivery methods pose significant limitations for inducing rich haptic sensations. First, physical contact with a paper or a pad induces a sensation of touch in addition to the sensations induced by the chemical. Therefore, controlling or studying the formation of sensations induced purely by the chemical is not possible. Second, as the skin remains covered by the paper or pad, the chemicals cannot be combined with other technologies for inducing haptic sensations, such as mechanical vibration or friction from moving on a textured surface. Therefore, inducing or studying haptic sensations beyond a single chemical solution at a time is not possible. Third, the chemicals are only delivered at the specific location of the pad and for the duration that the solution on the pad carries. Therefore, more dynamic application both temporally (e.g., sustaining the delivery of the chemical for a longer time, stopping the delivery after a small quantity) and spatially (e.g., delivering the chemical in a very small or a larger region, dynamically moving the point of delivery) is impossible. These constraints limit the study and applications of chemical stimulants for rich haptic sensations.

To overcome these limitations and unlock new possibilities for chemical haptics, we propose the use of ultrasound as a novel delivery and modulation mechanism for haptic chemicals. Acoustophoresis uses acoustic radiation forces exerted by sound waves, such as ultrasound, to suspend objects and liquids in mid-air (i.e., “levitate”)^12^. This same technology is also capable of stimulating the skin mechanically through vibration of the skin through ultrasonic pressure. Recent advances in acoustophoresis have enabled the spatiotemporal manipulation of liquids^13–15^, even in the presence of sound-scattering objects^16^. By leveraging the unique advantages of acoustophoresis, we can achieve contactless, interactive, and dynamic delivery of liquid chemicals onto the skin.

We study whether the chemical stimulants delivered by ultrasound acoustophoresis to the skin can induce haptic sensations and whether the pure sensation can be modulated by simultaneous mechanical stimulation by ultrasound through three human-subject studies. In the first study, ultrasound acoustophoresis is used for the contactless delivery of four chemical stimulants shown in previous work to induce sensations with delivery via cotton pads: menthol, capsaicin, cinnamal, and ethanol. The chemicals were dissolved in ethanol. In the second study, we deliver cinnamal using ultrasound acoustophoresis and also apply ultrasonic haptic feedback at the point of application. In a third study, we only applied ultrasonic haptic feedback to compare pure haptic feedback with the combination of chemical and haptic feedback. All studies follow a similar data collection procedure consisting of participants rating the perceived intensity on a 10cm visual analogue scale ranging from “no intensity” to “maximum imaginable intensity” for five minutes. After five minutes had passed, the participants also described the sensation they experienced by selecting words from a given list. Overall, the studies show that chemical stimulants delivered to the skin with acoustophoresis are perceivable and that the mechanism used for delivery—ultrasound—can be used to modulate the perception.

## Results

**Fig. 1:**
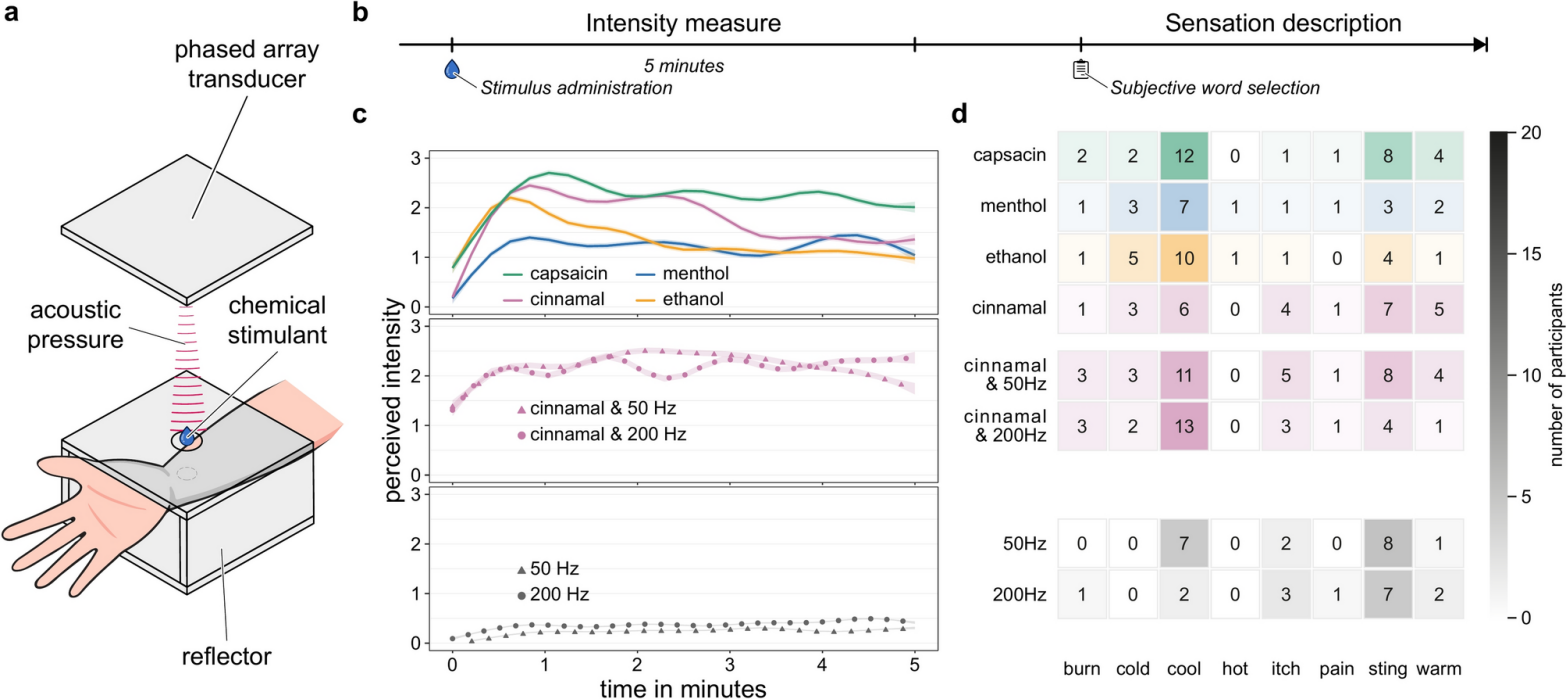
Illustration of the experimental setup, design, and results. (**a**) Experimental setup. The chemical was applied to the forearm using acoustic levitation. (**b**) Experimental protocol. Participants were asked to rate the perceived intensity of any given sensation for five minutes on a visual analogue scale (VAS, 0-10 cm), where 0 represented no intensity and 10 represented the maximum imaginable intensity^7^. After reporting on their perception, participants selected a set of words that described sensations perceived during the five minutes. (**c**) Plots of the GAMM smooths of the perceived intensity of haptic sensations and 95% pointwise confidence intervals for the smooth. The curve indicates the trend in the intensity data. *(top)* Perception of ethanol, capsaicin, cinnamal, and menthol; *(middle)* perception of cinnamal in combination with 50 Hz and 200 Hz acoustic stimulation. Smooth for the perception of cinnamal added for reference; and *(bottom)* perception of 50 Hz, and 200 Hz acoustic stimulation. (**d**) Frequency plots of the words participants selected to describe any sensation perceived during the study. *(top)* Words related to the perception of capsaicin, cinnamal, ethanol, and menthol; *(middle)* words related to the perception of cinnamal in combination with 50 Hz and 200 Hz ultrasound stimulation; and *(bottom)* words related to the perception of 50 Hz and 200 Hz ultrasound stimulation.

We invited a total of 160 participants across three pre-registered studies to report on the perceived intensity of any kind of sensation of a stimulus over time. The studies were conducted with identical apparatus (Fig. 1a) and procedure (Fig. 1b); however, they were conducted with varying stimuli. The first study investigated the perception of four solutions of chemical stimulants: ethanol (the solvent), capsaicin, cinnamal, and menthol. The second study investigated the perception of one chemical (cinnamal) in conjunction with an acoustic stimulus modulated to 50 Hz and 200 Hz, respectively. The third study investigated the perception of an acoustic stimulation modulated to 50 Hz and 200 Hz, respectively, alone.

**Fig. 2:**
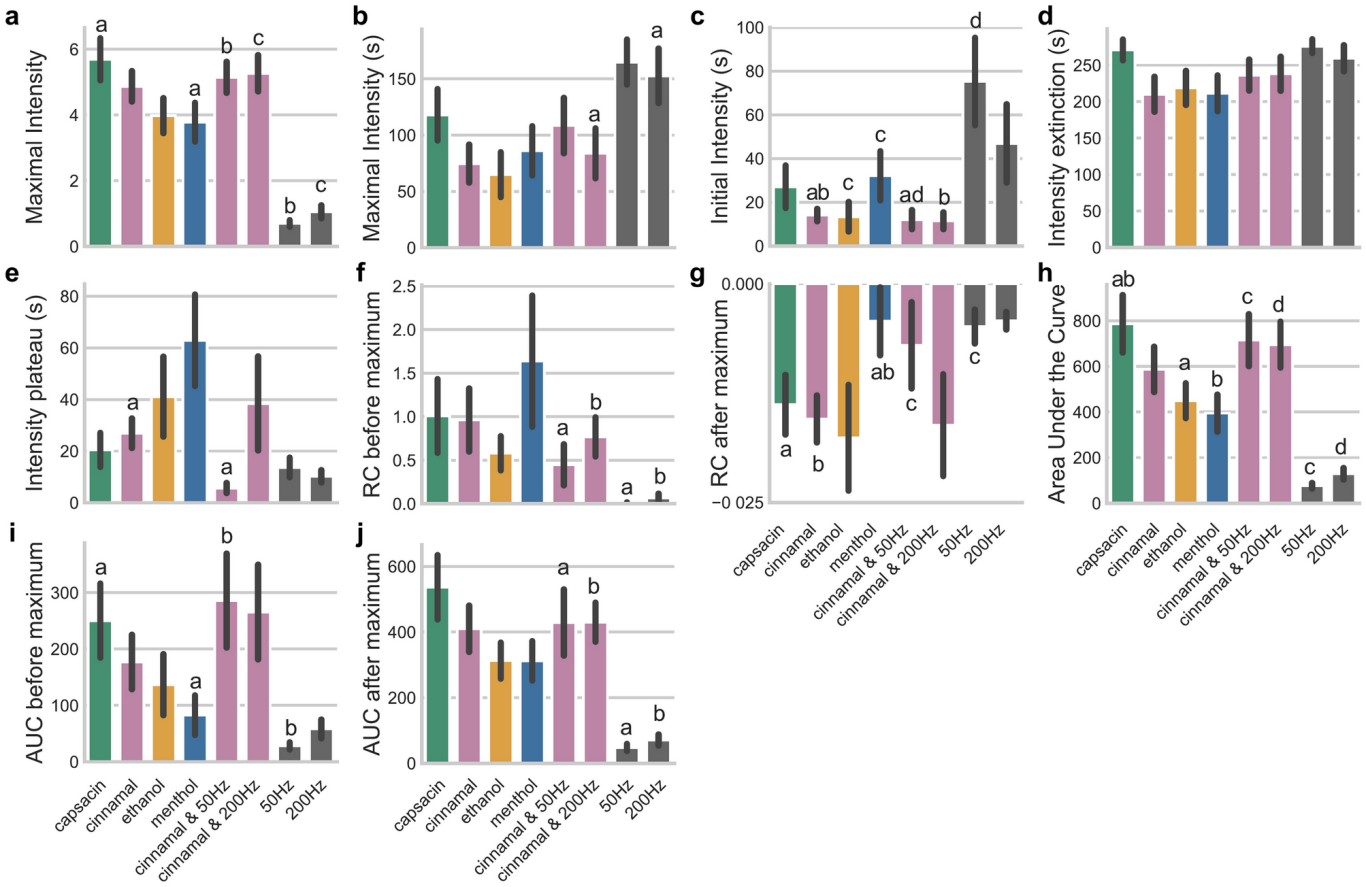
Time-intensity analysis for all three studies. (**a**) The maximal reported intensity. (**b**) The time in seconds at which the maximal intensity occurs. (**c**) The time in seconds at which the stimulant was initially perceived. (**d**) The time in seconds at which the stimulant was not perceived any more. (**e**) The number of seconds around the maximal intensity at which the reported intensity plateaued. (**f**) The rate of change before the maximal intensity occurred. (**g**) The rate of change after the maximal intensity occurred. (**h**) The area under the mean curve. (**i**) The area under the mean curve before the maximal intensity occurred. (**j**) The area under the mean curve after the maximal intensity occurred. Mean area with *same* letters indicate a significant difference at $p<.05$. Error bars represent standard errors of the means.

We analyse the data using a generalised additive mixed model (GAMM)^17^. To evaluate the model, we employ the Wald test^18^, allowing us to draw inferences on the perceivability of the stimuli. To draw inferences from the frequencies of words used to describe the sensation, we employ a $\chi ^2$ test, determining the difference in frequency within and between conditions. In addition, as an exploratory measure, we use time-intensity analysis (TI)^19^ to compare stimulus intensity perception between participants. We employed the Kruskal-Wallis test, with a Dunn post-hoc analysis, to determine significant differences. Figure 2 shows an overview of the TI analysis. A statistical significance was defined as $p<.05$.

### Chemical perception

The majority of participants reported perceiving the chemical stimulant applied to their skin; for capsaicin, 17 participants out of 20 reported perceiving any sensation from the stimulus, and for cinnamal, menthol, and ethanol, 18 reported so. The GAMM smooths (Fig. 1c, top) show the temporal development of the perceived intensity of each chemical stimulant. We found that all four chemicals are perceivable (ethanol: $W(8.82)=169.3, p<.001$; capsaicin: $W(8.69)=140.8, p<.001$; cinnamal: $W(8.84)=285.9, p<.001$; and menthol: $W(8.62)=62.5, p<.001$). However, the degrees of freedom and the Wald statistic are relatively high due to the large perceptual variance between participants.

The capsaicin condition was perceived stronger than the menthol and ethanol conditions: The maximal perceived intensity was significantly higher in the capsaicin condition ($M=5.70, SD=2.64$) than in the menthol condition ($M=3.78, SD=2.51; p=.046, CL=0.70$) and the time to the initial perception in the ethanol condition ($M=13.35s, SD=28.81s$) was significantly lower than the menthol condition ($M=32.09s, SD=47.65s; p=.026, CL=0.28$).

In addition, we found that the total AUC was significantly higher for the capsaicin condition ($M=786.90, SD=520.30$) than for the ethanol ($M=449.66, SD=325.30; p=.039, CL=0.29$) and menthol conditions ($M=394.90, SD=345.76; p=.014, CL=0.75$).

Participants described their perception after reporting intensity. Figure 1d shows the frequency of words used by the participants for sensations felt throughout the five-minute span. We found that the distribution of words is similar across all chemical conditions, except for the menthol condition. Overall, the word ‘cool’ was used to describe the perceived sensation across most chemical conditions, followed by the word ‘sting’; however, there are no significant differences. Anecdotally, some participants described the sensation as ‘tingling’, ‘tickling’, and ‘vibrating’.

Overall, capsaicin is perceived as the strongest but comparable to cinnamal. The perceived strength generally weakens over time.

### Perception modulation

Applying cinnamal in conjunction with acoustic stimulation is perceivable (50Hz: $W(8.83)=33.97, p<.001$ and 200Hz: $W(8.26)=66.25, p<.001$). The perception of cinnamal is strengthened through acoustic stimulation (Fig. 1c, middle).

Overall, acoustic stimulation elicited a higher perceived intensity at the moment of onset and sustained it for longer. The perceived intensity in the cinnamal & 50Hz condition is 0.39 units ($SE =0.01$) higher than the cinnamal condition, $t(19)=27.18, p<.001$, while the cinnamal & 200Hz condition is 0.45 units ($SE =0.01$) higher, $t(19)=30.85, p<.001$.

The time to the initial perception in the cinnamal & 50Hz ($M=12.05s, SD=19.08s; p=0.028, CL=0.29$) and cinnamal & 200Hz conditions ($M=11.52s, SD=17.23s; p=0.034, CL=0.30$) was significantly shorter compared to the pure cinnamal condition ($M=14.12s, SD=12.04s$).

The time in which the perceived intensity plateaus around the maximal perceived intensity is significantly higher in the cinnamal condition ($M=26.92s, SD=24.35s$) than in the cinnamal & 50Hz condition ($M=5.71s, SD=8.56s; p=0.003, CL=0.21$).

There is a significant difference in the distribution of words used to describe the sensation elicited by cinnamal and the sensation elicited by cinnamal & 200Hz, $\chi ^2 (8, N=20)=26.08, p=0.001$. This leads to the inference that the acoustic stimulation enhanced the ‘cool’ sensation. We observed a similar trend for cinnamal & 50Hz; this difference is, however, not significant, $\chi ^2 (8, N=20)=3.05, p=0.931$.

Overall, these results suggest that added acoustic stimulation shortens the time till perception and increases the time at peak perception of cinnamal.

### The effect of acoustic stimulation

Acoustic stimulation was perceivable (50Hz: $W(8.46)=121.20, p<.001$ and 200Hz: $W(8.76)=144.80, p<.001$). The smooth of the perceived sensation of the 50Hz condition is 1.93 units ($SE =0.01$) lower than the cinnamal & 50Hz condition, while the 200Hz condition is 1.84 units ($SE =0.01$) lower than the cinnamal & 200Hz condition. We found that the maximal perceived intensity in the cinnamal & 50Hz condition ($M=4.89, SD=2.32$) is significantly higher than in the 50Hz condition ($M=0.63, SD=0.46; p<.001, CL=0.99$). The same is true for the cinnamal & 200Hz condition ($M=5.27, SD=2.47$) and the 200Hz condition ($M=0.90, SD=0.84; p<.001, CL=0.94$).

The time to the initial perception in the cinnamal & 50Hz condition ($M=12.05s, SD=19.08s$) is significantly lower than the 50Hz condition ($M=75.24s, SD=84.89s; p<.001, CL=0.13$). The total AUC was significantly higher for both corresponding pairs of conditions. Cinnamal & 50Hz condition ($M=715.07, SD=497.29$) has a higher AUC than in the 50Hz condition ($M=76.54, SD=52.17; p<.001, CL=0.98$), and cinnamal & 200Hz condition ($M=695.59, SD=450.77$) has a higher AUC than in the 200Hz condition ($M=129.13, SD=104.50; p<.001, CL=0.92$). For the AUC before the maximal perceived intensity, the cinnamal & 50Hz condition ($M=285.96, SD=363.46$) is higher than in the 50Hz condition ($M=28.21, SD=26.91; p=.004, CL=0.77$). The area after the maximal perceived intensity was also significantly higher for both corresponding pairs of conditions. The cinnamal & 50Hz condition ($M=429.10, SD=440.30$) is higher than in the 50Hz condition ($M=48.33, SD=45.96; p<.001, CL=0.90$), and the cinnamal & 200Hz condition ($M=429.99, SD=266.70$) is higher than than in the 200Hz condition ($M=70.89, SD=70.30; p<.001, CL=0.95$).

Participants described the sensations differently between the cinnamal & 50Hz and 50Hz conditions ($\chi ^2 (8, N=20)=97.71, p < .001$) and the cinnamal & 200Hz and 200Hz conditions ($\chi ^2 (8, N=20)=25.72, p < .001$). In Fig. 1d, we see that the two chemical conditions are rated ‘cool’, ‘itch’, and ‘sting’ more often.

Overall, we found the acoustic perception alone to be perceived as weak, yet noticeable. Acoustic stimuli alone are perceived significantly less on almost all TI parameters compared to the chemical and acoustic stimulation counterpart.

## Discussion

We have demonstrated that chemical stimulants delivered by ultrasonic acoustophoresis can be perceived. The application of ultrasound to the chemicals modulates the perceived intensity of the delivered sensation. Furthermore, the combination of ultrasound and chemicals can speed up the onset of the sensation, increase the perceived intensity at the onset, and maintain the sensation for longer.

We show empirically that droplets of capsaicin, cinnamal, menthol, and ethanol produce a perceivable sensation when applied to the human skin. The perception is characterised to be generally weak yet consistently present among participants. The temporal behaviour of the perception is comparable to previous work; it peaks within the first minute after application and steadily decreases after the peak. The reported intensity is low compared to previous work; Højland et al.^7^, for instance, reported a moderate peak intensity for cinnamal ($M=5.18, SD=0.32$).

However, the intensity is surprisingly high considering the smaller quantity and administration method. The standard method of application through cotton pads allows for better control of quantity and evaporation^3,4,7^ compared to administration through acoustophoresis. We suspect that uncontrolled evaporation has led to the high frequency of participants reporting the sensation of feeling ‘cool’, in particular, since the solvent, ethanol, is known to feel cold on the skin due to evaporation^20,21^. Menthol and capsaicin, which are commonly said to feel cold and hot, respectively, were not rated high for those words; however, this is consistent with previous studies^3^ where capsaicin may also feel cool to certain individuals. Cinnamal being rated high for ‘itch’ is also consistent with previous studies^6,7^. Thus, the novel delivery mechanism does not appear to change the qualities of the perception.

The addition of ultrasonic stimulation to the chemical stimulant cinnamal led to a sharp rise at the beginning of the intensity curve. The intensity curve for pure ultrasonic feedback also starts from zero. Though the graph’s peak does not change, the area under it is greater than the area for the sum of the individual sensations. The reason for this phenomenon requires further studies. It is also opposite to the phenomenon of reduction in sensation when chemical and mechanical stimuli are combined^11^. We found that the 200Hz signal was not significantly different than the 50Hz signal. This does not match our expectations, given these signals target different receptors. The exact relationship between the ultrasonic wave frequency and its effect on the chemical stimulus still needs further investigation. This relationship may vary for the different stimulants, and hence, future studies are needed to investigate different stimulants.

Delivering chemical stimulants to the skin by ultrasound acoustophoresis presents new opportunities for haptics compared to other delivery mechanisms, such as manual application using cotton pads or wearable chemical haptic devices ^22^. First, acoustophoresis enables dynamic mixing of tactile stimuli. For example, we showed that chemical stimuli can be mixed and modulated by mechanical stimuli, namely ultrasound haptics. Similarly, different chemical stimuli could be mixed with each other as a cocktail, or acoustophoresis could add other types of mechanical stimuli, such as levitating particles and shooting those on the skin^23,24^. Second, acoustophoresis enables temporal modulation of the chemical stimuli. We demonstrated that a single droplet can be perceived and showed that this perception changes over time. For instance, the stimuli could be applied multiple times and their frequency modulated, such as to strengthen or elongate the sensation with multiple droplets. Third, acoustophoresis enables spatial modulation of the stimuli. We applied a single droplet onto a single location on the skin. However, the droplets could be applied simultaneously or sequentially to multiple locations on the skin or atomised to spread a single droplet over a larger area. Combined, these three opportunities could be used to create dynamic mixtures of chemical sensations on the skin or to study the formation of the sensations (e.g., the thermal grill illusion by applying hot and cold inducing chemicals spatially next to each other, alternating temporally them on the same location, or as a mixture).

These opportunities are promising, yet further research is required to refine the shown results. Our methodology of continuous intensity reporting allows for more fine-grained reporting on temporal development than previous work. However, the method does now allow for qualitative categorisation of perception while the solution is affecting perception. An option for further refinement of the sensory qualities of the chemicals is to employ the Temporal Check-All-That-Apply (TCATA) methodology, in which multiple sensory qualities are measured over time^25^. Such data, in combination with the data collected in our studies, could yield insights into the changing sensory qualities over time.

Overall, our findings show the first demonstration of digital control of chemical stimulation for haptic feedback. Such digital control allows for the psychophysical study of the free nerve endings in the skin, whose function and activation are not fully understood^26,27^. Similarly impactful are our findings for research within human-computer interaction, providing insights into the use of chemical stimulants for interactive technology. The use of acoustophoresis as a delivery mechanism for chemical stimulants has the potential for substantial advancements in future research and applications.

Ultimately, we conclude that chemical stimulants applied to the skin are perceivable and have the potential to be modulated interactively. As shown in previous work, these chemicals differ in intensity and sensory qualities, suggesting that our methodology of applying chemicals

In this paper, we contribute a novel way of administering chemical stimulants through acoustophoresis, yielding opportunities to study psychophysical phenomena without the use of confounding factors, such as cotton pads. In addition, we show that the perceived intensity of cinnamal can be modulated through ultrasonic pressure, applied to the skin. Our results contribute to a better understanding of the perception of chemical stimulants and act as an extension of the work conducted by Green and colleagues^1–5^.

## Methods

We ran three studies with an identical apparatus and procedure, however, changing the stimulus. The experiments were approved by the local ethics committee at the University of Copenhagen (504-0376/23-5000) and were conducted in accordance with the Declaration of Helsinki (2013). All participants provided informed consent before engaging with the study. All studies were pre-registered at osf.io/a9erb.

### Apparatus

**Fig. 3:**
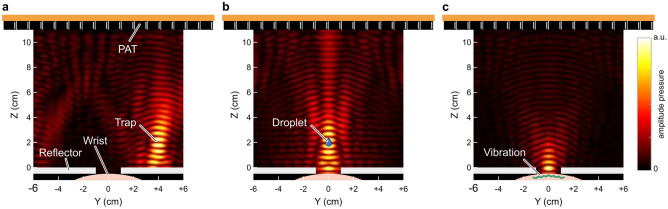
Simulation of the sound field while levitating a droplet and stimulating the human skin. (**a**) The simulated amplitude pressure field generated by the phased array transducer (PAT) focused at a single point to form a trap, able to suspend materials in mid-air above a reflector covering the participants wrist. (**b**) The same simulated trap moved and suspending a liquid droplet above the wrist. (**c**) The simulated amplitude pressure field generated to cause vibration in the wrist to stimulate the skin.

We built a device capable of acoustic levitation and ultrasound stimulation, both of which are facilitated through the same phased-array transducer (PAT). The PAT is comprised of $16\times 16$ Murata MA40S4S transducers (40 KHz, 10.5 mm diameter ($\approx$ 1.2 $\lambda$), delivering $\approx$ 8.1 Pa at 1 m distance when driven at 20 Vpp), which is commonly adopted in modern acoustophoretic systems^14,16,23,28^. We applied 20V to generate a safe ultrasound field for public use. The PAT dynamically updates the ultrasound signal that produces a focal point of acoustic radiation pressure that can be utilised to suspend and manipulate both solid and liquid materials in mid-air (see Fig. 3)^14,16^. When the focal point is directed to the human skin, the pressure amplitude is increased and modulated to the activation range of the mechanoreceptors in the skin, the focal point becomes perceivable as a vibrotactile sensation^29^. With this system, we can sequentially levitate materials and stimulate the human skin dynamically.

The device is based on the top-sided PAT arrangement and scattering levitation solver^16^, which allows for the creation of levitation points with a sound-scattering object in the sound field, contrary to most acoustophoretic systems that only allow empty levitation space to avoid sound-field distortion by external sound-scattering objects. This solver, however, requires geometrical information of the scattering object to pre-compute the levitation scattering contribution from the object. This process is, however, not real-time. Thus, it was not feasible to model each participant’s forearm as a scattering object. Instead, we place a hollow box made by acrylic sheets (see Fig.1a) as a fixed and solid scattering reflector, with a hole with 2 cm diameter on top to allow chemical droplets to fall onto the forearm, and to also enable amplitude-modulated stimulation to occur at the focal point. We implemented the scattering levitation solver based on the hardware and software framework provided by the OpenMPD platform^28^.

During the study, a drop of a chemical stimulant was injected 2 cm above the reflector at a distance of 4 cm to the centre of the hole in the reflector. The key to manipulating liquid droplets by the acoustophoretic system is not only to make the acoustic radiation force overcome gravity but also to adjust the ratio of acoustic radiation force to the interfacial force of droplet to avoid droplet atomization^16,30^. Once the chemical stimulant was successfully injected and suspended at the injection site, it was transported to the target hole area by continuously updating the levitation points. Upon reaching the designed location, it was then released to administer chemical and ultrasound stimulation to the skin.

### Procedure

We conducted three studies in sequential order. The studies differ only in the stimulus participants received. Both followed a between-subject design, such that each subject would evaluate the sensation of one stimulus condition to avoid confounding factors of a chemical stimulant being perceived for an extensive amount of time. A Latin square design determined the order of stimuli administered between subjects. All subjects received written and oral information about the experiment before giving their written consent. To counteract acoustic levitation’s novelty effect, the experimenter demonstrated the levitation capabilities using a 2mm polystyrene bead and answered the subjects’ questions.

Subjects were seated in front of the acoustophoretic device such that their right arm could reach inside the hollow reflector. Shortly after reaching into the reflector and placing their arm at the correct position, the experimenter administered the stimulus on the volar aspects of the subject’s forearm. Subjects were asked to assess the perceived intensity of any given sensation on the forearm on a visual analogue scale (VAS 0-10 cm) on a tablet computer, where 0 presents no intensity at all and 10 the maximal imaginable intensity^7^. The subject would adjust the scale continuously over a period of five minutes (see *Supplementary Information* for reasoning). The perceived intensity was recorded with a sampling frequency of 60 Hz.

After the five minutes had passed, subjects were asked to select any words from a list related to their perceived sensations over the period. Subjects could select none and write additional words in an open-ended format if they wished to. The listed words were presented in random order and with a description adapted from Green and Flammer^3^. Words and descriptions are listed in the *Supplementary Table S1*.

### Study 1: chemical perception

The first study investigated the temporal perception of chemical stimulants applied to the skin.

#### Participants

We recruited a total of 81 subjects for the first study (38 female, 42 male, range 18-50y, $M=25.75$, $SD=5.26$) primarily from the student body at local universities and social media posts as paid volunteers. One subject was excluded, as they did not follow the study protocol. We recruited only participants who reported no impairments or chronic or current pain in the right volar aspects of the forearm and no known food allergies. The room temperature was on average $23.70^{\circ }\text {C} \pm 1.17^{\circ }\text {C}$, while the surface skin temperature of the subjects was on average $36.28^{\circ }\text {C}$ $(SD=0.33^{\circ }\text {C})$. See *Supplementary Information* for sample size considerations.

#### Stimulation

In this study, we stimulated participants with one of four chemical stimulants soluted in ethanol: cinnamal (5% solution by volume^7^), menthol (40% solution weight by volume^6^), capsaicin (0.25% solution by weight^3^), or ethanol (carrier). Additional details are listed in *Supplementary Information Table S2*.

### Study 2: modulation of perception

The second study investigated the potential effect of ultrasound stimulation on the temporal perception of chemical stimulants on the skin.

#### Participants

We recruited a total of 40 subjects for the second study (19 female, 21 male, range 20-35y, $M=25.93$, $SD=3.58$) primarily from the student body at local universities as paid volunteers. We recruited only participants who reported no impairments or chronic or current pain in the right volar aspects of the forearm and no known food allergies. The room temperature was on average $23.15^{\circ }\text {C}$ $(SD=0.93^{\circ }\text {C})$, while the surface skin temperature of the subjects was on average $36.34^{\circ }\text {C}$ $(SD=0.38^{\circ }\text {C})$. See *Supplementary Information* for sample size considerations.

#### Stimulation

In this study, we used cinnamal (5% solution by volume^7^) as a chemical stimulant (see *Supplementary Information* for reasoning). After the application of the chemical, we stimulated the participant’s skin with an amplitude-modulated focal point^29^, vibrating the skin with either a 50Hz or 200Hz modulation, constantly over the five-minute study period. The focal point was directed through the hole in the reflector to reach the participants skin. These modulation frequencies stimulate two specific mechanoreceptors in the skin: the Meissner corpuscle with a peak vibration sensitivity at 50Hz and the Pacinian corpuscle with a peak vibration sensitivity at 200Hz^26,31^. These mechanoreceptors previously shown potential for producing reliable, dynamic haptic stimuli^29^.

### Study 3: acoustic perception

The third study investigated the potential effect of ultrasound stimulation on the wrist.

#### Participants

We recruited a total of 40 subjects for the second study (15 female, 24 male, one non-binary, range 21-57y, $M=29.77$, $SD=7.56$) primarily from the student body at local universities as paid volunteers. We recruited only participants who reported no impairments or chronic or current pain in the right volar aspects of the forearm. The room temperature was on average $22.46^{\circ }\text {C}$ $(SD=0.89^{\circ }\text {C})$, while the surface skin temperature of the subjects was on average $36.37^{\circ }\text {C}$ $(SD=0.22^{\circ }\text {C})$. See *Supplementary Information* for sample size considerations.

#### Stimulation

We stimulated the participant’s skin with an amplitude-modulated focal point^29^, vibrating the skin with either a 50Hz or 200Hz modulation, identically to the second study.

### Data analysis

We used a GAMM^17^ to model the intensity data and used a $\chi ^2$-test to test for differences in word distributions across conditions, as pre-registered. As an exploratory analysis, we conducted TI analysis^19^ to gain detailed insights into the perception of the stimulation.

The GAMM was fitted in R using the mgcv-library^18^ and the formula gamm(intensity $\sim$ stimulant + s(time, by = stimulant), data = data). We conducted the $\chi ^2$-test using the SciPy package in Python^32^ and the function scipy.stats.chisquare, comparing the word distributions.

We report on the intensity curve through TI analysis. We analyse the maximally perceived intensity across participants (Fig. 2a) and the time at which the maximal intensity occurs (Fig. 2b). We analyse the time of initial perception, the first time the intensity value exceeded 5% (the significance level) of the maximal intensity (Fig. 2c), and the time of the extinction of perception, the first time the intensity value falls below 5% of the maximal intensity after the maximal intensity occurred (Fig. 2d). Next we analyse the plateau around the maximal intensity, i.e., time duration around the maximal where the measured intensity is greater than 95% of the maximal intensity (Fig. 2e). In addition, we report the slope of a linear fit on the intensity values from the initial to the maximal intensity and from the maximal to the extinction in Fig. 2f and Fig. 2g, respectively. Last, we report on the area under the curve (AUC), both in total (Fig. 2h), before the maximal intensity (Fig. 2i), and after the maximal intensity (Fig. 2j).

## Supplementary Information

Supplementary Information 1.

Supplementary Information 2.

## Data Availability

Data and source code are available at osf.io/a9erb.
