# Supplementary material for: Ultrasound can deliver chemical stimulants to the skin and modulate their perception
Source: Sci Rep. 2025 Mar 25;15:10297. doi: 10.1038/s41598-025-94463-7 (PMC11937339; doi:10.1038/s41598-025-94463-7)
Supplement: Supplementary file 1 — Supplementary Information 1. [file 41598_2025_94463_MOESM1_ESM.pdf]

Ultrasound Can Deliver Chemical Stimulants to the Skin and Modulate Their Perception

Tor-Salve Dalsgaard[1,*], Arpit Bhatia[1], Lei Gao[2], Ryuji Hirayama[2], Sriram Subramanian[2], Joanna Bergström[1], and Kasper Hornbæk[1]

[1]Department of Computer Science, University of Copenhagen, Universitetsparken 1, 2100 Copenhagen
[2]Department of Computer Science, University College London, Euston Road 169, London
[*]torsalve@di.ku.dk

Supplementary Information

Sample size consideration

McKeown and Sneddon [1] describe a study design similar to ours, although they investigate the emotional valence of video clips. They suggest that 20 to 30 participants per condition would have been sufficient for their study to achieve a large effect size when using generalised additive mixed models (GAMMs) for analysis.

Pilot studies

Determining study duration

Subjects were asked to rate perceived intensity during a five-minute window. This duration was found suitable in a pilot study conducted prior to the first study. We recruited a total of 12 subjects for the pilot study (6 female, 6 male, range 22-32y, $M = 27.25$, $SD = 3.05$). We recruited only participants who reported no impairments or chronic or current pain in the right volar aspects of the forearm and no known food allergies. The room temperature was on average 23.42°C ($SD = 0.47$°C), while the surface skin temperature of the subjects was on average 36.37°C ($SD = 0.42$°C).

We administered a drop of either menthol, capsaicin, cinnemal, or ethanol to the volar forearm of the subjects. Subjects were asked to indicate two points in time by pressing a button: when they started perceiving sensations different from their normal perception and when they stopped perceiving sensations different from their normal perception. On average, subject stopped perceiving menthol after 94.80s (SD=54.00s), capsaicin after 888.61s (SD=1342.22s), cinnemal after 195.64s (SD=117.02s), and ethanol after 31.80s (SD=13.89s). Note that the capsaicin condition was heavily influenced by one participant who perceived a sensation for roughly 40 minutes. Such a long effect was not observed during any of the following studies. We decided on the five-minute window for rating the perceived intensity of the chemical stimulants as $> 90\%$ of subjects perceived the stimulation for shorter than five minutes.

Selecting a candidate stimulant for study 2

Subjects of study 2 were administered with cinnamal in conjunction with ultrasound stimulation, due to the results of a pilot study. We recruited a total of 24 subjects for the pilot study (8 female, 12 male, range 23-44y, 29.17 ± 4.73). We recruited only participants who reported no impairments or chronic or current pain in the right volar aspects of the forearm and no known food allergies. The room temperature was 22.39°C ± 0.58°C, while the surface skin temperature of the subjects was 36.60°C ± 0.17°C.

Based on the results of the first study, we selected cinnamal and capsaicin as candidates for our modulation technique using ultrasound stimulation. Thus, we conducted a between-subject study with two independent variables, chemical stimulant and ultrasound modulation frequency, similar to the following study 2 described in the main paper. The chemical stimulant variable had two levels, cinnamal and capsaicin, and the ultrasound modulation frequency was either 50Hz or 200Hz. We used a GAMM [1,2] to model the perceived intensity and found the effect of modulating cinnamal to be more promising, as the perceived intensity of the cinnamal & 50Hz was 0.43 units higher than the capsaicin & 50Hz and the cinnamal & 200Hz was 0.80 units higher than the capsaicin & 200Hz.

Movies

Movie S1. Video preview of the apparatus, procedure, and results of the study.

Table S1. The list of words shown after each trial for the subjects to describe their sensations during the five-minute trial period. Subjects could select any number of words to describe their sensations. The words were shown with a description of the word [3]. Subjects had the option to select none and describe sensations in their own words.

Sensation	Description
Itch	The sensation associated with a desire to scratch.
Sting/prick	Sharp sensations similar to those produced by an insect bite or a pin-prick, which may be constant or intermittent.
Burn	The sensation produced by extreme temperatures or chemical irritants, which may or may not be associated with a thermal sensation (either hot or cold).
Pain	Any sensation that 'hurts'.
Cool	A moderately cold sensation; neither warm nor cold.
Cold	The sensation of an uncomfortable lack of warmth.
Warm	The sensation of a moderate degree of heat.
Hot	The sensation of great bodily heat.

Table S2. The used chemicals and their solutions. Capsaicin, menthol, and cinnamaldehyde are soluted in ethanol. We reference the papers using the same solution and the commercial distributor of the chemical.

Chemical	Purity	Concentration	Solution	Reference	Source
Capsaicin	>60.0%	0.25%	by weight	[3]	TCI Europe N.V., Zwijndrecht, Belgium; PN: M1149
Menthol	>98.0%	40.00%	weight by volume	[4]	TCI Europe N.V., Zwijndrecht, Belgium; PN: M0321
trans-Cinnamaldehyde	>98.0%	5.00%	by volume	[5]	TCI Europe N.V., Zwijndrecht, Belgium; PN: C0352
Ethanol	96.0%		by volume		Les Grandes Distilleries de Charleroi N.V., Jumet, Belgium

References

1. McKeown, G. J. & Sneddon, I. Modeling continuous self-report measures of perceived emotion using generalized additive mixed models. *Psychol. Methods* **19**, 155–174, DOI: 10.1037/a0034282 (2014).
2. Wood, S. N. Fast stable restricted maximum likelihood and marginal likelihood estimation of semiparametric generalized linear models. *J. Royal Stat. Soc. (B)* **73**, 3–36 (2011).
3. Green, B. G. & Flammer, L. J. Capsaicin as a cutaneous stimulus: Sensitivity and sensory qualities on hairy skin. *Chem. Senses* **13**, 367–384, DOI: 10.1093/chemse/13.3.367 (1988).
4. Namer, B., Seifert, F., Handwerker, H. O. & Maihöfner, C. TRPA1 and TRPM8 activation in humans: Effects of cinnamaldehyde and menthol. *NeuroReport* **16**, 955, DOI: 10.1097/00001756-200506210-00015 (2005).
5. Højland, C. R., Andersen, H. H., Poulsen, J. N., Arendt-Nielsen, L. & Gazerani, P. A Human Surrogate Model of Itch Utilizing the TRPA1 Agonist Trans-cinnamaldehyde. *Acta Derm Venereol* **95**, 798–803, DOI: 10.2340/00015555-2103 (2015).
